# Cooling in Surgical Patients: Two Case Reports

**DOI:** 10.1155/2014/230520

**Published:** 2014-01-15

**Authors:** Bibi F. Gurreebun, Christos S. Zipitis, Ngozi E. Edi-Osagie, Ian M. Dady, Axel Sylvan

**Affiliations:** ^1^Neonatal Unit, Royal Albert Edward Infirmary, Wrightington Wigan and Leigh NHS Foundation Trust, Wigan Lane, Wigan WN1 2NN, UK; ^2^Manchester Academic Heath Science Centre, The University of Manchester, 46 Grafton Street, Manchester M13 9NT, UK; ^3^Newborn Intensive Care Unit, Central Manchester University Hospitals NHS Trust, Oxford Road, Manchester M13 9WL, UK

## Abstract

Moderate induced hypothermia has become standard of care for children with peripartum hypoxic ischaemic encephalopathy. However, children with congenital abnormalities and conditions requiring surgical intervention have been excluded from randomised controlled trials investigating this, in view of concerns regarding the potential side effects of cooling that can affect surgery. We report two cases of children, born with congenital conditions requiring surgery, who were successfully cooled and stabilised medically before undergoing surgery. Our first patient was diagnosed after birth with duodenal atresia after prolonged resuscitation, while the second had an antenatal diagnosis of left-sided congenital diaphragmatic hernia and suffered an episode of hypoxia at birth. They both met the criteria for cooling and after weighing the pros and cons, this was initiated. Both patients were medically stabilised and successfully underwent therapeutic hypothermia. Potential complications were investigated for and treated as required before they both underwent surgery successfully. We review the potential side effects of cooling, especially regarding coagulation defects. We conclude that newborns with conditions requiring surgery need not be excluded from therapeutic hypothermia if they might benefit from it.

## 1. Introduction

Moderate induced hypothermia to 33-34°C via total body or selective head cooling has become standard care for term infants who have suffered peripartum hypoxic ischaemic encephalopathy (HIE), following the publication of randomised controlled trials (RCTs) [1] and meta-analyses [2] demonstrating its effectiveness in reducing the composite outcome of death or disability in these neonates. However, these RCTs excluded patients with significant congenital abnormalities or conditions requiring surgery [1, 2]. This was partly because of the possibility of skewing data if these children also had congenital brain abnormalities, but also because of concerns regarding the systemic effects of hypothermia that could potentially affect surgery in these neonates [3, 4].

We report two cases of children born with congenital conditions requiring surgery who were successfully cooled, stabilised medically, and then rewarmed before surgery.

## 2. Case Report 1

AB was born at term in a district general hospital in poor condition, requiring full cardiopulmonary resuscitation including adrenaline and bicarbonate boluses, before responding at 12 minutes of life. Her first blood gas was as follows: pH 6.8, pCO_2_ 4.2 kPa (31 mm Hg), bicarbonate 5.5, and base excess −28. She was admitted to the neonatal unit for continued care, where she was soon noted to have large amounts of blood-stained and bilious gastric aspirates. Her first X-ray displayed the classic double-bubble sign of duodenal atresia. Her coagulation screen was abnormal (APTT ratio 3.2, INR 5.0), her haemoglobin was normal, and her platelet count was low. She had extra Vitamin K and was transferred to the regional Neonatal Intensive Care Unit (NICU).

Local guidelines recommend the initiation of therapeutic hypothermia in term infants with an Apgar score under 5 or continued resuscitation at 10 minutes of life or metabolic acidosis with a pH under 7.00 or a base deficit greater than 16 mmol/L at one hour of life, in the presence of moderate-to-severe encephalopathy as defined by an altered state of consciousness, abnormal tone, or abnormal primitive reflexes. AB had cerebral function monitoring which was moderately abnormal. She was noted to be poorly responsive, with hypotonia and absent primitive reflexes. As the criteria for therapeutic hypothermia were satisfied, this was initiated at 5 hours of life and given for 72 hours. She was treated for seizures on day 3 and her cranial ultrasound on day 2 showed increased thalamic echogenicity. An electroencephalogram, done on day 4, showed moderate abnormalities consistent with HIE with no subclinical seizures.

She required ventilator support for 9 days and inotropic therapy with dopamine, dobutamine, and hydrocortisone for 4 days. She had intravenous antibiotics for presumed sepsis. She required further doses of vitamin K, fresh frozen plasma, cryoprecipitate, and platelets and her gastrointestinal bleeding resolved by day 4 of life although gastric aspirates remained bilious. She had renal impairment by day 3, with oliguria, hyponatraemia, and oedema. This was managed conservatively with fluid restriction and resolved. She remained on total parenteral nutrition. She was treated for a chest infection from day 6 with IV antibiotics, and her chest X-ray showed right upper lobe consolidation.

AB was deemed medically stable for surgery on day 16 and she underwent a duodenoduodenostomy, a Ladd's procedure for malrotation detected intraoperatively, and an inversion appendicectomy as per local protocol.

Postoperatively, AB had 2 days of prophylactic antibiotics. She had one episode of line-related sepsis on day 4 post-operatively, treated with intravenous antibiotics. She was started on a partially hydrolysed formula once her gastric aspirates were nonbilious. This was increased as tolerated. She stayed in hospital mainly for the establishment of feeds and was discharged after one month on full feeds.

Her cranial ultrasound findings resolved on repeat scan and she had a normal MRI scan of her brain before discharge. She passed her hearing screening and her ophthalmology review revealed no abnormalities. She has since been reviewed at the age of six months and one year and has been feeding and growing well, with no neurodevelopmental concerns. At one year, she had normal tone, power, and reflexes in all four limbs and had started walking and had a vocabulary of two words.

## 3. Case Report 2

CD was born at term with an antenatal diagnosis of left-sided congenital diaphragmatic hernia. She cried at birth and was electively intubated. However, she had poor breath sounds on the left and was requiring 100% oxygen. She became pale and bradycardic at 15 minutes of age. Her endotracheal tube was changed, leading to a transient improvement. By 25 minutes she became pale and bradycardic again. She received full cardiopulmonary resuscitation, including doses of adrenaline and sodium bicarbonate, for 10 minutes before a response was seen. She was transferred to NICU for further management.

Her first gas showed a mixed respiratory and metabolic acidosis with a pH of 6.7. She was paralysed and sedated with atracurium and morphine infusions. She had some abnormal seizure-like movements at 3 hours of life and cerebral function monitoring was moderately abnormal. The criteria for initiation of therapeutic hypothermia were satisfied in her case as well; therefore, after due consideration, this was started at 4 hours of life and continued for 72 hours.

CD was started on parenteral nutrition and intravenous antibiotics as per protocol. Her coagulation screen remained normal. She had a mild renal dysfunction with good urine output, which normalised by day 4. On day 4, she had an episode of accidental blood loss from her umbilical arterial catheter, followed by a profound desaturation with no bradycardia or hypotension, for which she received a blood transfusion. In the following day, she had a respiratory deterioration with a right upper lobe collapse and raised inflammatory markers. She required high frequency ventilation for 24 hours and was treated with intravenous antibiotics for 10 days.

By day 9, CD's ventilatory requirements were decreasing, her inflammatory markers were normalizing, and her blood pressure was stable off inotropes. She was deemed stable for surgery and underwent a correction of her congenital diaphragmatic hernia, insertion of a Hickman line, and an inversion appendicectomy.

Postoperatively, she remained ventilated and paralysed for 3 days, after which she was weaned off. Enteral feeds were started 5 days post-operatively, once her nasogastric aspirates resolved. These were increased slowly until she was fully on enteral feeds by day 24.

CD had an electroencephalogram on day 6 of life which showed changes consistent with mild HIE. These resolved on repeat testing. Her cranial ultrasound scan was normal and an MRI of her brain was attempted but unsuccessful due to movement artefact. She has since been reviewed at 6months and 1 year and has been feeding and growing well with no neurodevelopmental concerns. At one year, she had normal tone, power, and reflexes in all four limbs. She was cruising to furniture and bottom shuffling. She had developed a good pincer grasp and could eat finger food. She had no words yet but babbled.

## 4. Discussion

It has been established that therapeutic hypothermia increases survival rate without neurological deficit in term or near-term infants with HIE [1, 2]. RCTs to date have excluded patients born with congenital abnormalities requiring surgery [1, 2] partly because of concerns regarding the potential side effects of hypothermia that could affect the outcome of surgery [3, 4].

Induced hypothermia has been shown to cause a reduction in heart rate, a decrease in blood pressure and cardiac output, and prolonged PR, QRS, and QT intervals on electrocardiograms [3]. However, these tend to resolve with rewarming and have not been shown in RCTs to relate to an increased risk of arrhythmias or increased need for inotropes [1, 2].

Similarly, hypothermia can cause a theoretical rise in lactate levels, increased risk of pulmonary hypertension, hypoxia, hyperglycaemia, electrolyte imbalances, and susceptibility to infection. However, none of those have translated into significant clinical risks in RCTs [3].

More importantly for surgical cases, hypothermia has been associated with deranged blood clotting [3–5]. This is caused by decreased platelet number and function and deranged fibrinolysis and clotting enzyme function [3]. Extreme hypothermia has previously been linked with an increased risk of pulmonary and intracranial haemorrhage [4]. However, in RCTs, moderate hypothermia has not been shown to be associated with a prolonged prothrombin or partial thromboplastin time, nor with increased risk of bleeding [1, 2]. There is an association between hypothermia and lower platelet counts, but not with an increased need for platelet transfusions [3]. It still remains important to check coagulation profiles in infants undergoing hypothermia, especially if they have an increased risk of haemorrhage, including conditions needing surgery.

Patient AB developed disseminated intravascular coagulopathy secondary to HIE and had signs of bleeding. Inducing hypothermia at a time of bleeding and an already deranged clotting can potentially exacerbate this. However, this was appropriately and effectively managed until the clotting and bleeding improved and surgery was not planned until after medical stabilisation. The effect of hypothermia on coagulation can therefore be negated in children requiring surgery if this is undertaken after rewarming and medical stabilisation.

There have also been concerns about hypothermia delaying wound healing and predisposing patients to wound infections [5]. However, this tends to be related to intraoperative hypothermia. In our case report, both patients underwent surgery several days after rewarming and neither had such problems.

We found one previous report of a neonate who underwent surgery for a tracheoesophageal fistula with concurrent therapeutic hypothermia. In this case as well, no issues were reported with wound healing or wound infections [5]. This patient also had a coagulopathy and required fresh frozen plasma during hypothermia. At the same time, hypothermia is often used during cardiac surgery with few adverse effects. These illustrate the potential for using hypothermia despite the need for concurrent surgical procedures.

Our two cases illustrate a different approach which is delaying surgery until after medical stabilisation, therapeutic hypothermia, and rewarming in children who may need both. In all such cases, the pros and cons of hypothermia against delaying surgery need to be weighed carefully. While this may not be possible for all surgical cases, our cases illustrate that newborns with conditions requiring surgery can in some cases have their surgery safely postponed until after medical stabilisation, therapeutic hypothermia, and rewarming.
